# Trends in Etiology and Mortality in Severe Polytrauma Patients with Traumatic Brain Injury: A 25-Year Retrospective Analysis

**DOI:** 10.3390/jcm14196986

**Published:** 2025-10-02

**Authors:** Olga Mateo-Sierra, Rebeca Boto, Ana de la Torre, Antonio Montalvo, Dolores Pérez-Díaz, Cristina Rey

**Affiliations:** 1 Department of Neurosurgery, Gregorio Marañón University Hospital, Complutense University of Madrid, 28007 Madrid, Spain; 2 Gregorio Marañón Health Research Institute, Complutense University of Madrid, 28007 Madrid, Spain; crey@salud.madrid.org; 3 Department of Neurology, Gregorio Marañón University Hospital, Complutense University of Madrid, 28007 Madrid, Spain; rebeca.boto@salud.madrid.org; 4 Department of Neurology, Alcorcón Foundation University Hospital, Complutense University of Madrid, 28922 Madrid, Spain; anadela.torre@salud.madrid.org; 5 Department of Neurosurgery, Burgos University Hospital, 09006 Burgos, Spain; anmontal90@gmail.com; 6 Department of Emergency Surgery, Gregorio Marañón University Hospital, Complutense University of Madrid, 28007 Madrid, Spain; lolaperezdiaz@hotmail.com

**Keywords:** trauma epidemiology, traumatic brain injury, trauma mortality

## Abstract

**Background:** Polytrauma remains a leading cause of mortality and disability worldwide. Although trauma-related deaths have declined in recent decades, the drivers of this trend remain incompletely understood. Traumatic brain injury (TBI) is the principal cause of death and long-term disability in polytrauma, making it a critical determinant of outcomes. This study aimed to examine long-term trends in clinical characteristics, management strategies, and outcomes of polytraumatized patients with TBI (PTBI), with a particular focus on factors influencing overall and cause-specific mortality. **Methods:** We conducted a retrospective observational study of a prospectively maintained trauma registry over a 25-year period (1993–2018) at the Gregorio Marañón University General Hospital (Madrid, Spain). Adult patients with PTBI were included. Epidemiological, clinical, and outcome data were analyzed globally and across four time periods. **Results:** Among 768 patients with PTBI, mean age was 43 years (±20), and 29% were female. Most sustained closed TBIs (96%) with concomitant severe injuries to the head, chest, and extremities (median Injury Severity Score [ISS] 27; median New Injury Severity Score [NISS] 34). Emergency surgery was required in 51%, and 84% were admitted to intensive care. Over time, the incidence of polytrauma decreased, mainly reflecting fewer traffic-related injuries following advances in prevention and legislation. Despite an increasingly older and comorbid population, ISS/NISS and early mortality declined, largely due to improvements in prehospital care and hemorrhage control. Although crude TBI-related mortality appeared unchanged (28%), this pattern likely reflects offsetting influences, including an older and more comorbid patient population, a higher relative burden of severe cases, and the limitations of mortality alone to capture gains in functional outcomes. **Conclusions:** Advances in trauma systems and preventive policies have substantially reduced the burden of polytrauma and improved survival. However, severe TBI remains the principal unresolved challenge, highlighting the urgent need for innovative neuroprotective strategies and greater emphasis on functional recovery.

## 1. Introduction

Trauma is a major global public health challenge and one of the leading causes of death worldwide [1], accounting for over 5.8 million deaths annually and a substantial proportion of fatalities among individuals under 45 years of age [2]. According to the World Health Organization (WHO), the main causes include road traffic accidents (RTAs), suicides, and homicides [3]. In Spain, trauma remains a frequent cause of morbidity and mortality, mainly due to RTAs, falls, and high-energy impacts, although these patterns have changed over time [4].

Although the global health landscape was profoundly shaped by the COVID-19 pandemic [5], declines in trauma-related morbidity and mortality had already been documented prior to its onset [6]. These improvements were largely attributable to advances in medical care and the centralization of services in specialized trauma centers [1,7]. In addition, legislative and technological interventions—particularly those targeting traffic safety—contributed to declining incidence and mortality [8,9].Together, these factors underscore the need to assess how the clinical and epidemiological profile of patients with severe trauma has evolved in specialized centers over the long term.

Polytrauma is defined as injury involving multiple body regions with two or more major lesions [10,11,12]. Trauma is considered “major” when the Injury Severity Score (ISS) or New Injury Severity Score (NISS) exceeds 15, both widely used metrics that correlate directly with case fatality rates (CFRs) [11,13]. Trauma remains a critical global health burden, ranking as the third leading cause of death worldwide and a predominant cause of morbidity and mortality in individuals younger than 40 years [1].

Within this context, traumatic brain injury (TBI), emerges as the key prognostic determinant, accounting for most trauma-related deaths and long-term disability [13,14,15,16,17]. Prognosis is further influenced by injury mechanism (e.g., RTAs, suicide attempts, assaults), physiological status at presentation [3,18,19], and the availability of specialized trauma care [20,21,22]. Notably, patients with comparable ISS or NISS may exhibit divergent outcomes [23,24,25,26], highlighting the need for refined prognostic models such as TRISS, CRASH, and IMPACT, which show stronger correlations with mortality but require complex analyses [25,26,27,28].

Mortality after severe trauma has declined significantly over recent decades [2,7,11], largely owing to early, protocolized management of massive hemorrhage—the foremost preventable cause of death, responsible for up to 40% of trauma fatalities [6,29,30,31,32]. In contrast, the CFR from TBI remains persistently high. Each year, TBI affects an estimated 50 million people worldwide [15,17,33,34], representing the leading cause of acquired brain injury among young adults. Prognosis worsens in the presence of hypotension or concurrent hemorrhagic injuries, underscoring the need for timely, high-quality multidisciplinary care. Preventive legislation [8,9] and advances in stabilization protocols [2,17,34,35] have further contributed to changes in the epidemiology and outcomes of TBI in recent decades.

Despite these advances, robust longitudinal evidence on polytrauma in Spain remains scarce. To date, the only prior work is the pilot study by Chico-Fernández et al. in 2016 [4]. The present study addresses this gap by providing long-term data on the incidence, causes, and prognostic factors of severe polytrauma with TBI (PTBI) based on a large single-center cohort spanning 1993–2018 at a level I trauma reference hospital in Madrid, Spain.

## 2. Materials and Methods

### 2.1. Study Design

This retrospective study analyzed prospectively collected data over a 25-year period prior to the COVID-19 pandemic. It included adult patients with severe traumatic brain injury (TBI), either as isolated trauma or as part of polytrauma, treated at the Emergency Department of Gregorio Marañón University General Hospital between June 1993 and October 2018.

### 2.2. Inclusion Criteria

- Adult patients (≥18 years);
- Polytrauma, defined as ISS > 15 or NISS > 15;
- Presence of TBI confirmed by clinical or radiological findings;
- Admission to our center within the study period (1993–2018);
- Received prehospital or in-hospital acute management;
- Complete medical records with accessible data.

### 2.3. Exclusion Criteria

- Patients under 18 years;
- Low-energy trauma or non-traumatic brain injuries (e.g., stroke, hypoxic encephalopathy);
- Isolated TBI without systemic polytrauma (ISS ≤ 15);
- Patients declared dead at the scene;
- Missing key variables or incomplete prehospital or in-hospital records;
- Other specific exclusions included patients with burns, hanging or drowning due to different pathophysiology and outcomes.

### 2.4. Variables Analyzed

The variables analyzed included epidemiological, clinical, prehospital and in-hospital parameters, as well as initial treatment, intensive care unit (ICU) admission, hospitalization, mortality, and complications. The study first evaluated the entire cohort and was subsequently stratified into four segments of nearly identical duration (75 months and 22 days each): Period 1 (24 June 1993–14 October 1999), Period 2 (15 October 1999–6 February 2006), Period 3 (7 February 2006–29 May 2012), and Period 4 (30 May 2012–7 October 2018).

Specific variables included:

- Epidemiological factors: age, sex, date of injury;
- Medical history and trauma characteristics: mechanism (blunt/penetrating), intent (assault, self-inflicted, or accidental);
- Trauma cause: traffic accidents (car, motorcycle, pedestrian), falls, assaults, others;
- Severity indicators and protective factors: seatbelt or helmet use, fall height, prehospital vital signs, emergency medical team care (SAMUR-061), initial shock, cardiopulmonary resuscitation (CPR), intubation, lactate, fluid resuscitation;
- In-hospital parameters: vital signs, emergency surgery, transfusions, injury location, Abbreviated Injury Scale (AIS), ISS, and NISS;
- Outcomes: ICU admission, complications, case fatality rate (CFR) (day 1, 30-day, overall), including preventable deaths.

### 2.5. Statistical Analysis

Continuous variables were expressed as mean ± standard deviation for normally distributed data, or as median and interquartile range for non-normally distributed data. Categorical variables were analyzed using Pearson’s Chi-square test with odds ratios (OR) and 95% confidence intervals (CI), or Fisher’s exact test when appropriate. Quantitative variables were compared using Student’s *t*-test for normally distributed variables and the Kruskal–Wallis test for non-parametric data. Binary logistic regression was employed for multivariate analysis. Survival analysis was performed using Kaplan–Meier survival curves, and Cox regression models were used to estimate hazard ratios (HR) with 95% confidence intervals. All analyses were conducted using IBM^®^ SPSS^®^ Statistics version 29, with *p* values < 0.05 considered statistically significant.

## 3. Results

A total of 2816 polytraumatized patients who met the study’s inclusion criteria were reviewed. Of these, 768 sustained concomitant polytrauma and TBI (PTBI) and constituted the analytic cohort.

### 3.1. General Analysis

Baseline characteristics are summarized in Table 1. The mean age of patients with PTBI was 43 years (±20); 29% were female and 71% were male. Most TBIs were closed injuries (96%). A substantial proportion of patients (53%) had no relevant medical history, 19% had one significant pre-existing condition, and 21% had two or more.

**Figure 1:**
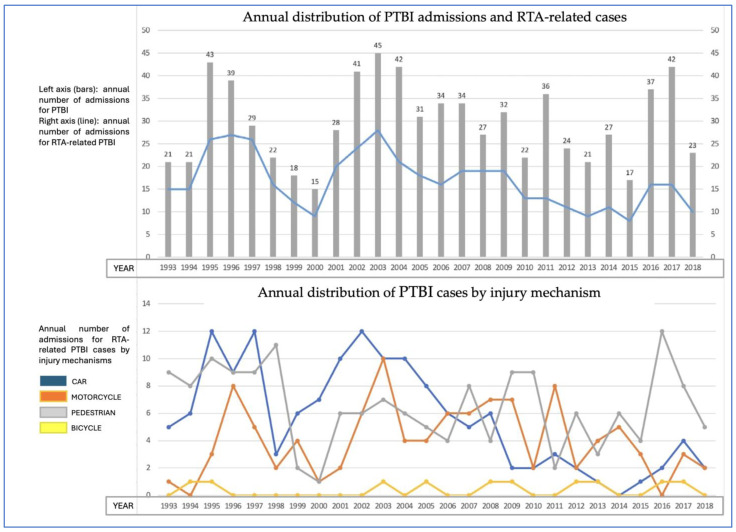
(**Top**): Annual distribution of PTBI admissions (bars) and RTA-related cases (line), 1993–2018. [Combined bar-and-line chart. The *X*-axis represents the study years (1993–2018). The *Y*-axis shows the annual number of cases. Bars indicate the total number of admissions per year, while the superimposed line denotes the subset of cases attributable to RTAs]. The chart illustrates the progressive decline in PTBI incidence, particularly reflecting reductions in RTA-related trauma. (**Bottom**): Annual distribution of PTBI cases by injury mechanism, 1993–2018. [The *X*-axis represents the study years. The *Y*-axis indicates the annual number of PTBI admissions per each mechanism. Colors represent mechanisms: car accidents (blue), motorcycle accidents (orange), pedestrian run-overs (grey), and bicycle-related trauma (yellow)]. A marked decline in RTAs, particularly car accidents (blue) was observed, while pedestrian-related trauma (grey) showed a progressive increase. Motorcycle-related trauma (orange) remained relatively stable, and bicycle-related trauma (yellow) rose modestly in later years.

Annual PTBI admissions fluctuated across the study period (Figure 1). RTAs accounted for the majority of cases (58%, *n* = 445), followed by falls from height (29%, *n* = 223), suicide attempts (5%, *n* = 38), assaults without weapons (4%, *n* = 31), and assaults involving sharp or firearm weapons (3%, *n* = 23). Over time, however, the frequency and distribution of RTAs shifted significantly (*p* < 0.001): car accidents predominated in the early years, whereas pedestrian injuries became increasingly common toward the end of the study. These temporal shifts paralleled changes in protective behaviors, particularly seatbelt and helmet use, as discussed later.

Most patients presented with severe associated injuries to the head, chest, and extremities, as reflected in AIS scores. The median ISS and NISS were 27 and 34, respectively. Overall, 51% required emergency surgery, half involving the CNS, and 84% were admitted to intensive care, with a median ICU stay of 8 days.

With respect to complications, 50% of patients did not develop clinically significant events and only 9% required reoperation. The overall 30-day CFR was 34%. Early fatality occurred in 8% on arrival and in 19% within the first 24 h. The median time to death was 14 days. The distribution of causes of death identified brain injury as the leading etiology (28%), followed by exsanguination (5%).

### 3.2. Initial Care

Prehospital care was provided in 94% of cases by 061-SAMUR emergency teams. Endotracheal intubation was required in 64% of cases, and CPR was performed in 5%. Initial shock was observed in 18% of patients, mainly due to CNS injury or hemorrhage (Table 1). Despite this, most patients arrived hemodynamically stable, although with severe neurological impairment: the median GCS was 7, with 52% of patients presenting with a GCS < 8, and 17% exhibiting pupillary abnormalities.

### 3.3. Period-Based Analysis

Patient characteristics were then analyzed across four equal time periods (Table 2). Mean age increased progressively from 38 to 54 years (*p* < 0.001). The proportion of female patients rose from 25% to 37% (*p* = 0.014), and comorbidities increased from 21% to 61% (*p* < 0.001). Beyond the variation in RTA-related admissions (*p* < 0.001), there was a marked rise in suicide attempts (0–13%, *p* < 0.001), as well as an increase in the proportion of patients managed by prehospital emergency services (89–96%, *p* = 0.016).

**Figure 2:**
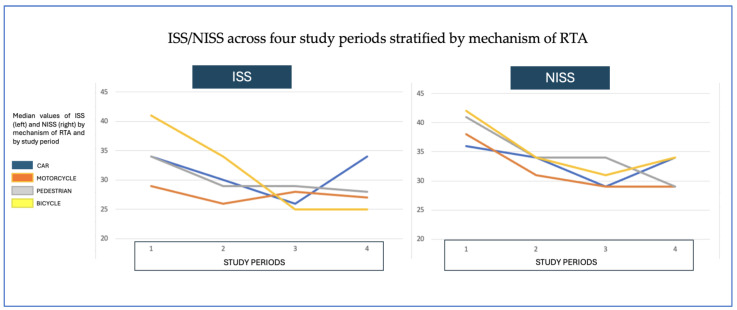
Distribution of median ISS values on the left and NISS values on the right across the four time periods of the study. [The *X*-axis represents the four study periods (1–4), and the *Y*-axis shows the absolute median values. Colors represent specific mechanisms: blue = car accidents; orange = motorcycle; gray = pedestrian (run-over); yellow = bicycle]. Both ISS and NISS demonstrated a progressive decline, indicating decreasing severity of PTBI over time.

Initial assessments revealed less severe neurological compromise over time, with median GCS improving from 3 to 11 (*p* < 0.001). Systemic condition also improved, with median ISS/NISS decreasing from 34/41 in the first period to 25/29 in the last (*p* < 0.001; Figure 2). In contrast, no significant differences were observed in the rates of emergency surgery, reintervention, or ICU admission.

Table 3 presents a detailed analysis of the most frequent causes of PTBI. Car accidents declined steadily as a cause of admission (from 52 to 12 cases per period), alongside increased adoption of safety measures—likely attributable to legislation and technological advances. In-hospital CFRs in this group, however, remained unchanged at 33%. By contrast, motorcycle-related trauma remained relatively stable in frequency (from 24 to 18 cases), but helmet use increased substantially. This correlated with improved neurological status at admission (median GCS from 4 to 11) and by a significant reduction in fatality.

### 3.4. Case Fatality Rates

Overall CFRs declined significantly from 1993 to 2018 (*p* = 0.039), including early mortality upon arrival (*p* < 0.001), mainly due to reduction in deaths from exsanguination and distributive shock, also known as vasodilatory shock. These improvements were statistically significant across periods and persisted after adjustment for ISS/NISS (*p* < 0.05), suggesting that survival gains were not solely attributable to milder injury profiles.

**Figure 3:**
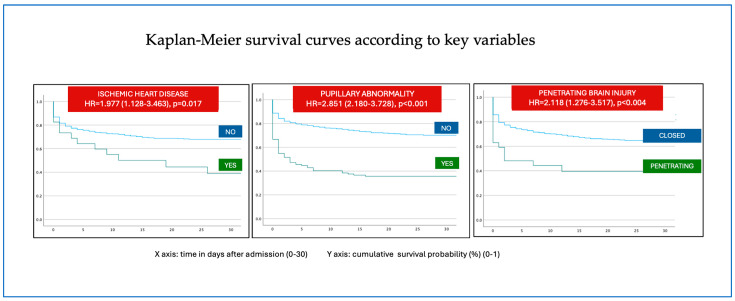
Mortality data according to key variables analyzed using Kaplan–Meier survival curves. Patient comorbidities, including ischemic heart disease, as well as penetrating trauma, pupillary abnormalities and CNS injury, were associated with significantly increased case fatality.

In contrast, CNS injury-related CFR remained unchanged at 28%. A progressive reduction in CFR was observed across the analyzed time periods. These findings are illustrated for selected variables in Figure 3 through Kaplan–Meier survival curves and summarized in Table 4.

Subgroups with the highest statistically significant CFRs are shown in Table 5. These included patients of advanced age (*p* < 0.001), those with ischemic heart disease (*p* = 0.017), mechanisms such as falls from height (*p* = 0.005) or firearm injury (*p* < 0.001), higher ISS/NISS values, penetrating trauma (*p* < 0.004), pupillary abnormalities (*p* < 0.001), and markers of severe systemic compromise such as hypotension, intubation, shock, or the need of CPR (*p* < 0.001). Similarly, patients requiring chest tube insertion (*p* < 0.001) or emergency abdominal surgery (*p* = 0.002) had higher CFRs.

Although CFRs related to hemorrhage and non-neurosurgical emergency interventions declined, CNS injury-related CFR remained unchanged across all analyzed periods (Figure 3), underscoring its persistent clinical impact.

## 4. Discussion

This study provides a comprehensive analysis of the incidence, causes, prognosis, and long-term variability of PTBI, drawing on one of the most extensive institutional trauma registries reported to date. The 25-year observation period allows for the identification of temporal shifts in patient profiles, injury mechanisms, management, and outcomes. Such longitudinal analyses remain scarce, particularly in southern Europe, and virtually absent when the focus is on PTBI, despite its relevance as the leading cause of death and disability in polytraumatized patients.

Our findings corroborate earlier epidemiological observations: PTBI primarily affects middle-aged adults (mean age ~40 years) and remains more frequent in men, although gender disparities have progressively narrowed over time [14,36]. Although the cohort remains predominantly male (71%) and relatively young (mean age 43), a gradual increase in patient age was observed across the study period. This trend reflects global demographic changes as noted by Palacio et al. (2024), who emphasized that aging populations are increasingly vulnerable to trauma-related morbidity due to frailty, comorbidities and slower recovery trajectories [36].

In our study, 47% of patients had at least one comorbidity—most frequently hypertension, psychiatric disorders, and substance use—associated with higher mortality and complication rates, in line with Glynn et al. (2024) [37]. Pupillary abnormalities at admission, present in 17% of cases, emerged as early indicators of severity, supporting the prognostic value proposed by Veerapaneni et al. (2024), who demonstrated that pupillary dysfunction enhances outcome prediction in TBI patients [38]. Other admission-based predictors have also been identified, including early thrombocytopenia [39], abnormal serum magnesium or calcium levels [40], and the Stress Index [41], all of which independently correlate with mortality in severe TBI. These findings underscore the value of early physiological and laboratory markers to refine risk stratification. These findings underscore the value of early physiological and laboratory markers to refine risk stratification. Together, they highlight the opportunity to integrate clinical, laboratory, and frailty-based predictors into early decision-making algorithms for PTBI.

Importantly, these demographic changes—particularly the rise in older and frail patients with multiple comorbidities—may offset the expected benefits of advances in trauma and neurocritical care, thereby contributing to persistently high TBI-related mortality. Similar findings were recently reported by Huang et al. (2024), who showed that the Geriatric Trauma Outcome Score reliably predicts mortality in older TBI patients, highlighting how frailty and comorbidity strongly influence prognosis [42].

From a systems perspective, prehospital care was intensive and aligned with modern trauma guidelines [2,6,25]: 94% of patients received SAMUR-061 care, with high rates of intubation (64%) and CPR (5%). These practices reflect international standards emphasizing early hemorrhage control and structured assessment, as reviewed by Kim and Kim (2025) [43]. Emergency surgery was frequent (51%), and our data support the PROPHET study, which identified prehospital indicators (such as poor neurological status, hypotension, and intubation) as predictors of urgent in-hospital procedures [44]. Nonetheless, early mortality remained high—8% upon arrival and 19% within 24 h—mostly due to TBI (28%). These figures are consistent with those reported by Kapapa et al. (2025) and highlight the need for refined prognostic tools and individualized decision-making, particularly in older, comorbid, and neurologically compromised patients [45].

The period-based analysis revealed a steady increase in mean age from 38 to 54 years, consistent with population ageing and changing mechanisms of injury, as previously documented in the Scottish national series [14]. The prevalence of comorbidities rose in parallel, a trend with direct implications for physiological response to trauma and outcome, as confirmed in our cohort and in prior studies [2,18]. Interestingly, substance abuse rates remained stable, diverging from global patterns [10,19].

Another key factor is the shifting case-mix: while preventive strategies have reduced the number of milder trauma cases (particularly road traffic accidents), the proportion of patients with severe injuries—including high-grade TBI—has increased. This changing baseline may conceal real therapeutic advances, as the registry captures an increasingly complex patient population. Notably, when outcomes were adjusted for ISS/NISS, survival improved significantly over time, indicating that therapeutic progress occurred even though crude TBI-related mortality remained apparently static.

In terms of injury mechanisms, RTAs were the predominant mechanism overall, but their contribution declined substantially across the study period, whereas falls from height and suicide-related trauma became increasingly prominent [4,9,10,11,13]. Indeed, a striking decline in both car and motorcycle-related trauma (28% to 6%) was observed, most likely reflecting the impact of road safety legislation, mandatory seatbelt and helmet use, and stricter enforcement [8,9,46]. By contrast, pedestrian injuries showed a progressive rise, particularly among older adults in urban environments, now posing a major challenge for public health and city planning, described in European as well as in US contexts [47,48,49].

In fact, in US, for example, pedestrian deaths increased by 77% between 2010 and 2022, representing the highest levels in more than four decades [49,50]. Globally, pedestrians, together with cyclists and motorcyclists, continue to be among the most vulnerable groups in road traffic crashes, a risk further amplified by the proliferation of SUVs in urban areas and insufficient pedestrian-friendly infrastructure [48,49]. These findings emphasize the need for preventive strategies specifically targeting vulnerable road users. Measures such as the redesign of urban infrastructure (e.g., elevated crosswalks, improved street lighting), enforcement of lower speed limits in densely populated areas, safer vehicle design standards, and enhanced public awareness campaigns have been proposed to mitigate this growing challenge [48,49,50].

Alarmingly, suicide-related trauma rose sharply in recent years, accounting for 13% of cases in the final study period, in line with national mortality statistics (7.9 per 100,000 inhabitants in 2017). These findings highlight suicide as a growing public health emergency requiring targeted preventive strategies, as emphasized by national and international agencies (WHO) [51,52].

Over time, nearly all patients received specialized prehospital care, reflected in improved physiological status at hospital admission, including higher GCS scores and better systemic stability. This improvement likely contributed to the overall decline in early mortality. Outcomes were also influenced by treatment in a level I trauma center, where multidisciplinary expertise and standardized protocols have been consistently associated with improved survival. Schubert et al. (2019) reported lower mortality in US trauma-certified centers [1], while Moore et al. (2017) demonstrated an 18.2% relative reduction in risk-adjusted mortality in Canadian centers in 2012 compared to 2006 [6].

Our findings are in line with these international experiences, reinforcing the importance of regionalized trauma systems. Yet, when focusing specifically on severe TBI, the picture becomes more complex: our results diverge from some international reports that have shown modest reductions in mortality over time [14], while others describe stagnation despite advances in neurocritical care [16,34]. Our findings align more closely with these latter reports, in contrast with the Scottish national series, where modest mortality reductions were documented [14]. A similar variability has been observed in Latin American cohorts, such as the recent Mexican series by Martínez-Herrera et al. (2024), which identified context-specific mortality predictors and confirmed the persistent lethality of severe TBI despite systemic advances [53]. This heterogeneity likely reflects differences in trauma systems, ICU protocols, and the availability of rehabilitation resources.

In the present series, a significant decline in case fatality was observed, attributable to advances in hemorrhage control, modern transfusion protocols, and innovative treatment strategies—including surgical, endovascular, and hybrid room interventions [2,29,30,54]. As a result, exsanguination-related deaths declined markedly. However, case fatality related to CNS injury persisted at 28%, remaining the leading cause of death through the study and even showing a relative increase in later years. This paradox likely reflects the success of interventions targeting hemorrhage and shock, thereby unmasking the persistent lethality of severe TBI [13,15,16,17]. Furthermore, relying exclusively on mortality as the outcome may obscure meaningful progress in survival with disability. Functional outcomes, quality of life, and long-term neurological trajectories would provide essential context to fully assess advances in TBI care. In our registry, functional outcomes were not systematically collected, which limits the ability to determine whether survival gains were accompanied by disability.

These findings underline a persistent gap in trauma care: while systemic improvements have reduced preventable deaths, survival for patients with severe TBI has stagnated for decades. Addressing this challenge will require breakthroughs beyond current trauma protocols, integrating neuroscience, critical care, and public health strategies to truly alter the prognosis of TBI in polytrauma.

In summary, this study highlights four key findings:

1. The incidence of PTBI progressively declined, largely reflecting advances in road safety legislation and preventive strategies.
2. Patient demographics shifted toward an older and more comorbid population, a trend that may counterbalance therapeutic progress and complicate outcomes.
3. Overall case fatality decreased, mainly due to improvements in prehospital management, hemorrhage control, and systemic trauma care; however, changes in case-mix and hospital-level practices may influence these trends.
4. TBI-related fatality persisted at 28%, with this apparent stability likely reflecting an older, more comorbid population, a higher burden of severe cases, and the inherent limitations of crude mortality rates, reinforcing the need to emphasize functional outcomes and to develop targeted neuroprotective strategies.

The main strength of this study lies in its size, completeness, and length of follow-up, with nearly all PTBI cases admitted to a reference level I trauma center over 25 years systematically included. This provides a rare opportunity to assess temporal trends in incidence, management, and outcomes using prospectively collected data in a high-volume European center.

Limitations: This study has several limitations. Its retrospective design restricts the ability to control for confounders or infer causality. Functional and quality-of-life outcomes were not systematically collected, preventing a full assessment of survival with disability. The interpretation of “unchanged TBI-related mortality” should therefore be considered cautiously, as crude mortality rates may mask therapeutic progress. Finally, although the registry is one of the most extensive single-institution series in Southern Europe, its monocentric nature may limit generalizability to other trauma systems with different organizational structures and resources.

Future research should focus on prospective, multicenter registries with standardized collection of functional outcomes and rehabilitation data, which will be crucial to disentangle demographic effects from true therapeutic advances and to guide the development of innovative neuroprotective strategies.

## 5. Conclusions

This long-term study offers robust evidence that the incidence and case fatality of PTBI have evolved significantly over the past three decades. Preventive legislation and advances in trauma management have reduced overall mortality, even in an older and more comorbid patient population. Nevertheless, the persistently high case fatality from CNS injury underscores a major unresolved clinical challenge. These results emphasize the urgent need for innovative neuroprotective strategies, optimized prehospital and in-hospital neurological care, and targeted public health interventions to reduce the burden of TBI in polytrauma.

## Figures and Tables

**Table 1:** General characteristics of the patient cohort. Data are presented as number of cases (percentage) and median (interquartile range). SD: standard deviation.

Patients			Neurological Condition		
Age in years (mean, SD)	43	20	Initial assessment (n/%)		
Sex (n/%)			Pupillary abnormality	129	17
Female	220	29	Deficit	52	7
Male	548	71	INJURIES BY REGION/SEVERITY (median, IQR)		
Comorbidities (n/%)			Head AIS	4	3–5
0	409	53	Face AIS	0	0
1	145	19	Thorax AIS	2	0–3
≥2	158	28	Abdomen AIS	0	0–2
Type of comorbidities (n/%)			Extremities AIS	2	0–3
Hypertension	65	9	Skin AIS	0	0–1
Cardiopathy	23	3	ISS	27	19–38
Ischemic heart disease	23	3	NISS	34	24–41
Diabetes mellitus	32	4	INITIAL SURGERIES (n/%)		
Anticoagulation	33	4	Chest tube insertion	169	22
Substance abuse	38	5	Emergent surgery	388	51
Alcoholism	32	4	Neurosurgery	193	25
Psychiatric disorder	61	8	CASE FATALITY RATES (n/%)		
INITIAL MANAGEMENT (n/%)			Total (30 days)	262	34
061-SAMUR team	722	94	Death upon arrival	64	8
Prehospital status			Death on first day	137	19
Intubation	488	64	CAUSE OF DEATH (n/%)		
CPR	36	5	CNS injury	212	28
Apnea	115	15	Exsanguination	40	5
Shock	136	9	Sepsis	13	2
CAUSES OF SHOCK AT ADMISSION (n/%)			Multiorgan failure	13	2
CNS	36	26	Cardiac/Lung injury	14	2
Multiple	26	19	Distributive shock	5	0.7
Hemoperitoneum	20	15	COMPLICATIONS (n/%)		
Fractures	12	9	0	380	50
Other	42	31	1	252	33
No shock	632	82	>1	14	2

**Table 2:** Demographic, comorbidity, and trauma mechanism characteristics of polytrauma patients with TBI across four study periods. Data are presented as number of cases (percentage) unless otherwise indicated. SD: standard deviation.

Studyperiods	1	2	3	4
*n*	184	206	191	187
Age in years, mean (+/−SD)	38 (+/−17)	38 (+/−17)	42 (+/−21)	54 (+/−22)
Sex, *n* (%)				
Female	47 (25%)	55 (27%)	48 (25%)	70 (37%)
Male	137 (75%)	151 (73%)	143 (75%)	117 (63%)
Comorbidity, *n* (%)				
No history	145 (79%)	101 (49%)	91 (48%)	72 (39%)
Hypertension	0	6 (3%)	20 (10%)	39 (21%)
Cardiopathy	0	3 (1%)	6 (3%)	14 (7%)
Ischemic heart disease	0	7 (3%)	6 (3%)	10 (5%)
Diabetes mellitus	0	6 (3%)	6 (3%)	20 (11%)
Anticoagulation	0	6 (3%)	5 (3%)	22 (12%)
Substance abuse	6 (3%)	16 (8%)	8 (4%)	8 (4%)
Alcoholism	5 (3%)	14 (7%)	8 (4%)	5 (3%)
Psychiatric disorder	5 (3%)	16 (8%)	17 (9%)	24 (13%)
Mechanism of trauma, **n** (%)				
Car	52 (28%)	59 (29%)	24 (13%)	12 (6%)
Motorcycle	24 (13%)	31 (15%)	37 (19%)	18 (10%)
Bicycle	2 (1%)	2 (1%)	2 (1%)	5 (3%)
Pedestrian	59 (32%)	32 (16%)	49 (26%)	59 (32%)
Fall	40 (22%)	36 (17%)	53 (28%)	68 (36%)
Suicide attempt	0	6 (3%)	10 (5%)	25 (13%)
Firearm	4 (2%)	7 (3%)	5 (3%)	3 (2%)
Sharp weapon	1 (0.5%)	2 (1%)	4 (2%)	1 (0.5%)

**Table 3:** Main epidemiological changes in PTBI caused by traffic accidents across the four periods of time analyzed in this study. PTBI: polytrauma with traumatic brain injury; SD: standard deviation; GCS: Glasgow Coma Scale; IQR: interquartile range; ISS: Injury Severity Score; NISS: New Injury Severity Score; CNS: central nervous system.

	Car-Related PTBI				Motorcycle-Related PTBI			
Period	1	2	3	4	1	2	3	4
*n*	52	59	24	12	24	31	37	18
Age in years, mean (+/−SD)	34 (+/−14)	34 (+/−14)	26 (+/−8)	41 (+/−21)	24 (+/−6)	29 (+/−12)	33 (+/−13)	37 (+/−14)
Seatbelt/Helmet, *n* (%)	7 (13%)	18 (31%)	12 (50%)	9 (75%)	8 (33%)	11 (35%)	21 (57%)	16 (89%)
Trauma scores								
GCS, median (IQR)	5 (3–9)	8 (5–12)	7 (3–14)	6 (4–15)	4 (3–10)	6 (3–11)	7 (3–13)	11 (6–15)
IIS, median (IQR)	34 (24–50)	30 (19–38)	26 (20–34)	34 (26–36)	29 (22–42)	26 (20–36)	28 (22–24)	27 (18–34)
NISS, median (IQR)	36 (25–50)	34 (25–43)	29 (26–41)	34 (29–41)	38 (25–47)	31 (25–41)	29 (22–37)	29 (22–34)
Case fatality								
Total, *n* (%)	17 (32%)	18 (33%)	4 (15%)	4 (33%)	5 (20%)	9 (30%)	4 (11%)	2 (11%)
On arrival, *n* (%)	3 (6%)	1 (2%)	1 (4%)	0	0	0	1 (3%)	0
Due to CNS injury, *n* (%)	13 (24%)	13 (24%)	4 (16%)	4 (33%)	3 (12%)	9 (30%)	4 (11%)	2 (11%)

**Table 4:** Evolution of mortality along the study periods. IQR: interquartile range; GCS: Glasgow Coma Scale; ISS: Injury Severity Score; NISS: New Injury Severity Score; ICU: Intensive Care Unit; CNS: Central Nervous System.

Periods	1	2	3	4
n	184	206	191	187
Trauma scores, median (IQR)				
GCS	3 (3–9)	8 (3–12)	7 (3–13)	11 (6–15)
ISS	34 (25–50)	27 (17–36)	25 (19–34)	25 (16–34)
NISS	41 (29–50)	32 (22–41)	29 (24–38)	29 (22–38)
Injury distribution per region (AIS), median (IQR)				
Head	5 (4–5)	4 (3–4)	4 (3–4)	4 (3–5)
Face	0	0 (0–1)	0 (0–2)	0 (0–2)
Thorax	3 (0–4)	3 (0–4)	1 (0–3)	0 (0–3)
Abdomen	0 (0–2)	0	0 (0–2)	0 (0–2)
Extremities	2 (0–3)	2 (0–3)	0 (0–3)	0 (0–3)
Skin	0	0 (0–1)	0 (0–1)	0
Initial surgeries, *n* (%)				
Chest tube insertion	43 (23%)	44 (21%)	39 (20%)	43 (23%)
Emergent surgery	95 (52%)	114 (55%)	99 (52%)	80 (43%)
Neurosurgery	27 (15%)	58 (28)	57 (30%)	34 (18%)
ICU admittance, *n* (%)				
ICU	146 (79%)	181 (88%)	167 (87%)	147 (79%)
Case Fatality Rates, *n* (%)				
Total	75 (41%)	73 (35%)	58 (30%)	57 (30%)
On arrival	29 (16%)	10 (5%)	15 (8%)	10 (5%)
First day	61 (33%)	53 (26%)	26 (14%)	27 (14%)
Cause of death, *n* (%)				
CNS injury	52 (28%)	61 (30%)	47 (25%)	52 (28%)
Exsanguination	9 (5%)	14 (7%)	14 (7%)	3 (2%)
Sepsis	2 (1%)	6 (3%)	4 (2%)	1 (0.5%)
Multiorgan failure	3 (2%)	8 (4%)	1 (0.5%)	1 (0.5%)
Cardiac/lung injury	2 (1%)	8 (4%)	3 (1%)	3 (2%)
Distributive shock	1 (0.5%)	2 (1%)	2 (1%)	0

**Table 5:** Statistical analysis of case fatality rates based on different variables in multivariate analysis. HR: Hazard Ratio; CI: Confidence Interval; CPR: Cardiopulmonary resuscitation; GCS: Glasgow Coma Scale; ISS: Injury Severity Score; NISS: New Injury Severity Score.

Variable	*p*	HR	CI 95%
Patients			
Advanced age	<0001	1.01	1.004–1.016
Period 4	<0.001	0.806	0.717–0.906
Ischemic heart disease	0.017	1.977	1.128–3.463
Type of trauma			
Penetrating trauma	<0.004004	2.118	1.276–3.517
Motorcycle RTA	<0001	0.457	0.289–0.721
Fall from height	0.005	1.458	1.124–1.892
Fire weapon	<0.001	2.549	1.457–4.457
Initial assistance			
Intubation	<0.001	2.677	1.950–3.674
CPR	<0.001	6.323	4.344–9.202
Initial Shock	<0.001	2.808	2.161–3.648
Fixed pupil	<0.001	2.851	2.180–3.728
GCS	<0.001	0.82	0.790–0.851
Normal systolic pressure	<0.0101	0.988	0.984–0.991
ISS	<0.001	1.055	1.045–1.064
NISS	<0.001	1.058	1.049–1.067
Initial surgery			
Chest tube insertion	<0.001	1.544	1.185–2.011
Emergent surgery	<0.001	0.394	0.303–0.511
Limb surgery	<0.001	0.133	0.071–0.252
Abdominal surgery	0.002	1.785	1.231–2.588

## Data Availability

No new data were created.

## Abbreviations

AIS	Abbreviated Injury Scale
CFR	Case Fatality Rates
CNS	Central nervous system
CRASH	Corticosteroid Randomization After Significant Head Injury
GCS	Glasgow Coma Scale
IMPACT	International Mission for Prognosis and Analysis of Clinical Trials in TBI
ICU	Intensive Care Unit
ISS	Injury Severity Score
NISS	New Injury Severity Score
PTBI	Polytrauma with Traumatic Brain Injury
RTA	Road Traffic Accidents
SAMUR-061	Madrid Emergency Medical Service
TBI	Traumatic Brain Injury
TRISS	Trauma and Injury Severity Score
WHO	World Health Organization
